# Does mammary ductoscopy have a role in clinical practice?

**DOI:** 10.1186/1477-7800-3-16

**Published:** 2006-06-30

**Authors:** W Al Sarakbi, M Salhab, K Mokbel

**Affiliations:** 1 The Breast Care Centre, St. George's & The Princess Grace Hospitals, London, UK; 2 Consultant Breast & Endocrine Surgeon, St. George's Hospital, Blackshaw Rd, London SW17 0QT, UK

## Abstract

**Background:**

Mammary ductoscopy (MD) is a newly developed endoscopic technique that allows direct visualisation of the mammary ductal epithelium using sub-millimetre fiberoptic microendoscopes inserted through the ductal opening onto the nipple surface. These scopes also provide working channels for insufflation, irrigation, ductal lavage, and possible therapeutic intervention. MD can be performed under local anaesthesia in the office setting.

The objective of this study is to assess the technical feasibility of mammary ductoscopy, and examine its role in guiding ductal excision surgery and the early diagnosis of malignancy.

**Methods:**

Mammary ductoscopy (MD) was performed using a 1 mm fiberoptic microendoscope (Mastascope TM) in 26 patients (age range: 14–73 years): 13 patients undergoing mastectomy (n = 12) or lumpectomy (n = 1) for ductal carcinoma (including 12 cases of DCIS and one case of infiltrating ductal carcinoma) and 13 patients with pathological nipple discharge (PND) and benign breast imaging and simple discharge cytology. Of the latter group: 10 procedures were performed under local anaesthesia (LA) in the office setting and 3 procedures were carried out under general anaesthesia (GA) to guide duct excision surgery. The ductoscopic appearances in this group were graded between 0 and 5 (D0–D5) according to the degree of suspicion.

**Results:**

Intraoperative MD was accomplished in 11 (84.6%) of 13 patients undergoing surgery for DCIS. MD was unsuccessful in 2 cases: one patient (aged 73 years) had sclerosis of the nipple and one patient had preoperative vital blue injection in the subareolar region as part of the sentinel node biopsy thus resulting in inadequate visualisation. Intraductal pathology was visualised in 8 (80%) of the 10 cases undergoing mastectomy but ductoscopic cytology was positive for malignancy in only 2 cases (sensitivity = 16%, specificity = 100%). In the office setting, MD was accomplished in 9 (90%) out of 10 patients with PND and was well tolerated (mean pain score = 3.8 out of 10: range 0–7). Of these 10 patients; MD was inadequate (D0) in one patient due to complete occlusion of lumen by the lesion, showed a papilloma in 3 patients (D3), duct ectasia (D2) in 3 patients, irregular thickening of the lumen suspicious of DCIS (D4) in one patient and non-specific benign findings (D2) in 2 patients. Three women with benign ductoscopy and ductoscopy-assisted cytology were reassured and treated conservatively. The remaining 7 patients had ductoscopy-guided duct excision which revealed DCIS in one, papilloma in 4 and benign breast disease in 2 patients. Adequate cellular yield was obtained in 7(70%) out of 10 cases (benign cytology). The three patients who had MD under GA during microdochectomy had benign endoscopic appearances and final histology (one papilloma and 2 cases of duct ectasia).

**Conclusion:**

MD is technically feasible in most patients and has a potential in the early detection of breast cancer. The procedure can be performed safely in the office setting and should be considered in all patients presenting with a single duct PND. MD has the potential to reduce the number of duct excision procedures and minimise the extent of surgical resection. Ductoscopic cytology is not sufficiently sensitive for the diagnosis of malignancy and the development of a biopsy tool that obtains tissue under direct visualisation is required.

## Background

Mammary ductoscopy (MD) is a new endoscopic technique that has been evolving over the last 15 years [1-3].

**Figure 1:**
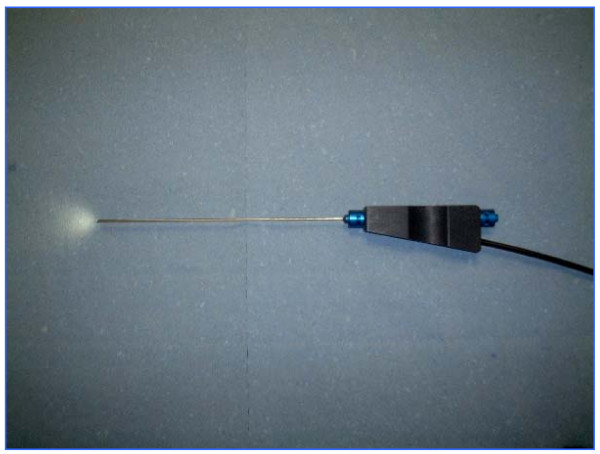
A breast ductoscope with a 1 mm external diameter and a 0.45 mm working channel.

**Figure 2:**
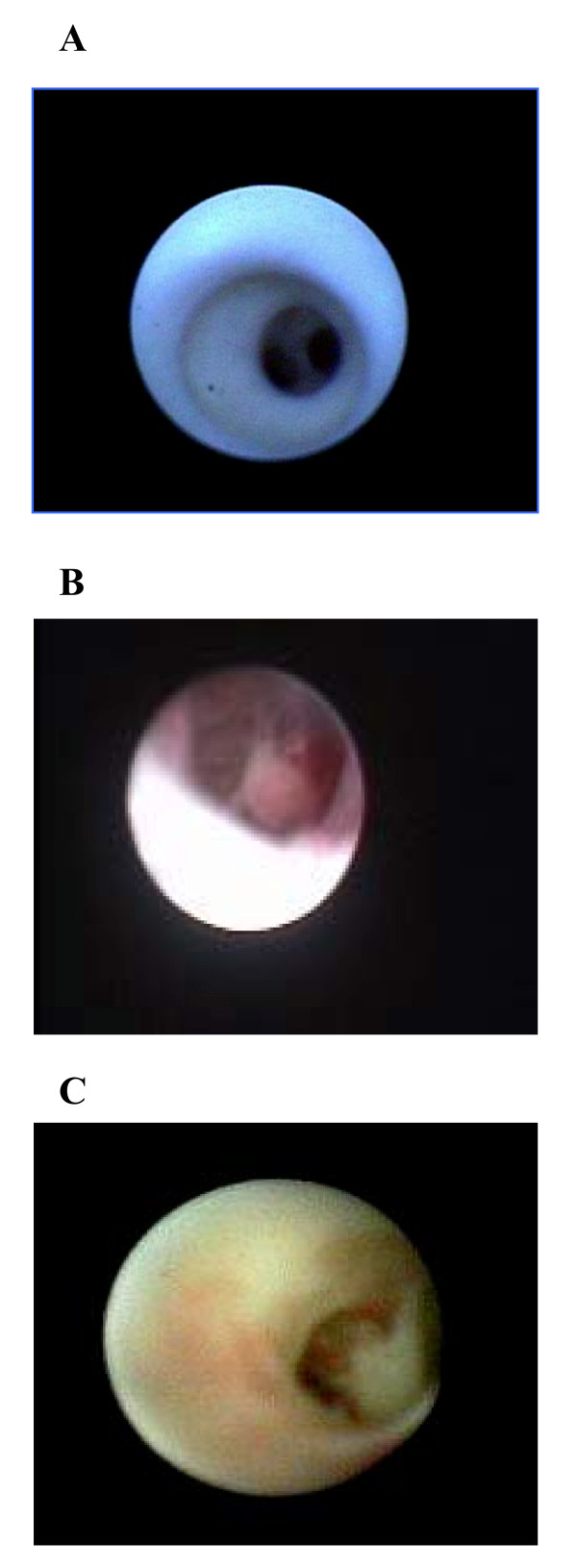
Mammary ductoscopy images: (A) Normal mammary ducts, (B) Intraductal papilloma (C) DCIS.

The initial scopes were of a large calibre with limited optics and lacked any working channels thus leading to a poor image quality [4]. Furthermore the images generated by the earlier scopes were too small and imprecise for accurate clinical judgement. Recent technological advances in endoscopic techniques, however, have overcome many of these obstacles. The current generation of microendoscopes (rigid or flexible) use excellent fibreoptics and measure between 0.7 mm and 1.2 mm in external diameter [2] (Figure 1). Inserted through the ductal opening on the nipple surface, they allow direct visualization of the mammary ductal epithelium where most benign and malignant pathologies originate [2,5]. These scopes also provide working channels for insufflation, irrigation, ductal lavage, and possible therapeutic intervention [2]. The sharp clear magnified images (Figure 2) viewed on a video monitor combined with the use of intraductal biopsy devices such as micro-brushes and other biopsy tools allow the retrieval of tissue specimens under direct visualisation for cytopathological analysis [2,5,6]. MD can be performed under local anaesthesia in the office setting with minimal discomfort and no reported complications [2].

The potential clinical applications of MD include the management of women with pathological nipple discharge, guiding of breast conserving surgery for cancer, and screening of high risk women [5,7-11].

PND is a relatively common symptom accounting for approximately 5% of all women attending symptomatic breast clinics [12]. Papilloma is the commonest pathological finding in women with PND accounting for 40% to 70% of cases followed by adenomatous or papillary epithelial proliferations (14%) [12,13]. However the incidence of malignancy (invasive or in situ) as a cause of PND varies between 1 and 23% depending upon the series studied [12]. The most reliable approach to both establish the diagnosis and control the discharge is ductal excision, the success of which is dependant on identifying the correct origin of the discharge. When a specific duct cannot be identified then blind excision of the retroareolar ductal system is usually performed.

Ductography is the traditional diagnostic technique used for investigating PND. Unlike ductography, ductoscopy can detect lesions that do not completely obstruct the ductal lumen. Furthermore, MD can detect multiple lesions within the same duct. It also plays an important role in guiding and minimising surgical duct excision as it offers accurate localization of the lesion, and can negate the need for surgery if the PND was proven to be benign on both endoscopy and biopsy [2]. Other advantages of MD include ductal lavage under direct visualization, and intraoperative guidance especially for lesions deep within the ductal system [10]. In addition to visualising intraductal lesions, cytological analysis of endoscopically retrieved ductal lavage has been recently reported to be more accurate than simple discharge cytology [1,3,12,14].

This study is part of an ongoing long-term research project aimed at exploring the potential clinical applications of MD.

## Patients and methods

MD was performed on a group of 26 patients (Age range: 14 – 73) after obtaining an informed consent.

We used a 1 mm fiberoptic microendoscope (Mastascope™). The endoscope was inserted through the ductal opening on the nipple surface after dilating the relevant duct with a suitable probe (Bowmann's lacrimal dilators). Saline solution or sterile water was injected into the duct through the working channel (0.45 mm) in order to widen it and facilitate the passage of the endoscope for clear visualisation of the intraductal space. The optical viewing and endoscopic system magnifies breast tissue up to 60 times its actual size and allows the identification of breast lesions up to 1/100^th ^the size of those detected with conventional mammography and magnetic resonance imaging (MRI). Distance of ductoscopic navigation ranged between 0 – 10 cm. The procedure can be performed under local anaesthesia (topical local anaesthetic cream plus intradermal local anaesthetic injection at the areolar margin) in the office setting [1,2,5].

MD was performed on a total of 26 patients divided into the following subgroups:

Group (A) consists of 13 patients undergoing mastectomy (n = 12) or lumpectomy (n = 1) for ductal carcinoma (including 12 cases of DCIS and one case of infiltrating ductal carcinoma). This group underwent intraoperative MD under general anaesthetic (GA) to assess the intraductal pathology. The tumour-bearing quadrant was massaged until nipple discharge was obtained for index duct identification.

Group (B) consists of 13 patients presenting with PND. Conventional investigations including mammography and/or ultrasound and simple discharge cytology were negative for malignancy. 10 of these patients underwent MD in the office setting to assess the cause and the presence of any suspicious pathology, and 3 patients underwent MD under (GA) to guide duct excision surgery.

Mamoscopic views of cancerous tissue included irregular thickening of the lumen, ulcerating proliferation, cross-bridging structures, extrinsic compression of ducts, and irregular polypoid masses. Both papillomas and duct ectasia had more characteristic and therefore typically diagnostic appearances (Figure 2). Papillomas appeared as smooth well-defined outgrowth, while duct ectasia appeared as dilated ducts with small solid white debris. These findings were confirmed by the subsequent histological analysis. The predictive value of these views is yet to be validated in large multi-centre prospective trials.

We proposed a new scoring system to describe ductoscopic appearances. According to this system the ductoscopic appearances were graded between 0 and 5 (D0–D5) according to the degree of suspicion (Table 1). This system complements but does not substitute the final histological validation.

**Table 1:** Proposed scoring system for ductoscopic appearances (Mokbel's classification system)

D0	Inadequate views/unsuccessful procedure
D1	Normal mammary ducts
D2	Typically benign appearance
D3	Low index of suspicion/indeterminate most likely benign
D4	Appearances suspicious of malignancy
D5	Appearances suspicious of malignancy plus positive ductscopic cytology

## Results

Intraoperative MD was accomplished in 11 out of 13 patients in group A (84.6%). MD was unsuccessful in 2 cases: one patient (aged 73 years) had sclerosis of the nipple and one patient had preoperative vital blue injection in the subareolar region as part of the sentinel node biopsy thus resulting in inadequate visualisation. Intraductal pathology was visualised in 8 (80%) of the 10 cases undergoing mastectomy but ductoscopic cytology was positive for malignancy in only 2 cases (sensitivity = 16%, specificity = 100%). In the single case of lumpectomy, the patient presented with a single nipple discharge. Mammography, ultrasound and simple discharge cytology showed no evidence of malignancy. Ductography revealed an intraductal papilloma. Initially, MD was performed under local anaesthetic (LA) and showed extensive epithelial proliferation suggestive of DCIS 5 cm from the nipple. MD guided excision showed low grade DCIS (clear margins). MD showed an intraductal papilloma in 3 patients' non-specific benign changes in one patient. Subsequent ductoscopy-assisted duct excision confirmed these endoscopic findings.

In the office setting MD was accomplished in 9 (90%) out of 10 patients with PND and was well tolerated (mean pain score = 3.8 out of 10: range 0–7). Of these 10 patients; MD was inadequate (D0) in one patient (due to complete occlusion of lumen by the lesion), showed a papilloma in 3 patients (D3), duct ectasia (D2) in 3 patients, irregular thickening of the lumen suspicious of DCIS (D4) in one patient and non-specific benign findings (D2) in 2 patients. Three women with benign ductoscopy and ductoscopy-assisted cytology were reassured and treated conservatively. The remaining 7 patients had ductoscopy-guided duct excision which revealed DCIS in one, papilloma in 4 and benign breast disease in 2 patients.

Adequate cellular yield was obtained in 7 (70%) out of 10 cases and interpreted as benign. Table 2 illustrates the correlation between ductoscopy appearances and post-operative histology in patients who underwent surgery, and cytology in patients' who were treated conservatively.

**Table 2:** Findings in patients undergoing MD in the office setting for pathological nipple discharge.

**Patients**	**Appearance**	**Classification**	**Histology**
1 (10%)	Inadequate	D0	Papilloma
3 (30%)	Papilloma	D3	3 Papilloma
3 (30%)	Duct ectasia	D2	None (conservative management) benign cytology
1 (10%)	DCIS	D4	DCIS
2 (20%)	Non-specific benign	D2	Benign breast disease

MD was also used to guide duct excision in the 3 patients undergoing surgery for PND under GA. All the three patients had benign endoscopic appearances and final histology (one papilloma and 2 cases of duct ectasia).

**Figure 3:**
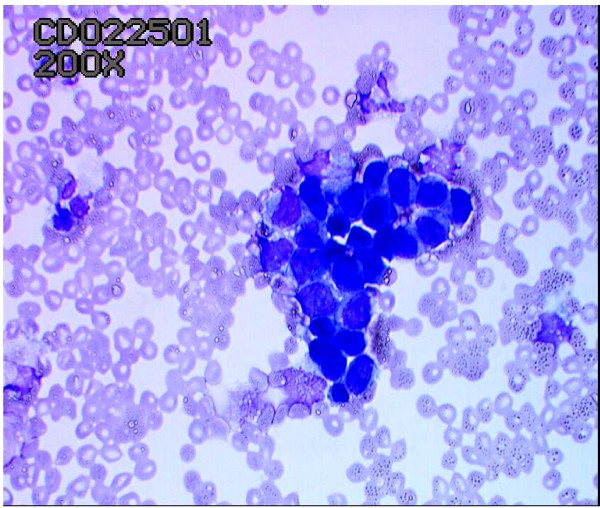
Ductoscopic cytology showing DCIS.

Although ductoscopic lavage cytology (Figure 3) revealed malignant cells in some cases of DCIS, overall it had a low cellular yield and lacked sensitivity (16%).

## Discussion

MD can be performed safely in the office setting and should be considered in all patients presenting with a single duct PND. The technique has the potential to reduce the number of duct excision procedures and minimise the extent of surgical resection. Simple light transillumination through the skin during MD can be used to guide duct excision. We have recently described a new technique involving ductoscopy and the use of a blue dye to perform microdochectomy [15,16]. MD can also potentially reduce the need to perform duct excision in patients with PND due to benign disease thus resulting in cost savings.

There are, however, recognised limitations to the use of MD in clinical practice. The first limitation is related to the breast anatomy. MD examines 1 – 2 ducts per breast and leaves the remaining 13 – 18 ducts that open at or just below the nipple surface unexamined. Although this allows access to the main central ducts draining 75% of the breast volume [17], it remains unknown whether these ducts are the commonest sites of malignancy [2]. Currently, MD is incapable of reaching the peripheral small branches of the ducts due to the scopes' diameter [2,5,18]. Thus it is unable to visualise the terminal duct-lobular unit (TDLU) where malignant lesions often originate. A recent report by Going *et al*. demonstrated the complex architecture of the nipple-areolar complex and the impending limitations of MD in the evaluation of the ductal system. The three-dimensional model of the nipple constructed by the authors revealed three distinct duct populations some not accessible to MD or ductal lavage [17]. The conclusion from this study is that accurate knowledge of breast duct anatomy in three dimensions is needed to understand the potential limitations of MD for breast cancer screening.

Future development of new smaller calibre micro-endoscopes that can be maneuovered to more peripheral sites may partially overcome this limitation [19].

The second limitation is related to histological verification of endoscopic findings. Current biopsy tools need re-evaluation, as the challenge of verifying endoscopic appearances with histological diagnosis has not yet been overcome. The development of reliable intraductal biopsy tools capable of obtaining tissue sample sufficient for histological diagnosis has been hampered by the small diameter of the working channels of the scopes. In addition, ductal washings during MD retrieves only one third of the fluid originally infused. Therefore, there is a considerable fraction of fluid and cells that remain trapped, probably within the smaller, more distally located ducts. Furthermore ductoscopic cytology frequently reveals atypia in cases of papilloma [20].

Previous studies showed that cytology samples obtained through MD-assisted ductal lavage is highly spicific, but it lacks the required sensitivity to predict whether the breast contains cancer [21,22]. Our study confirms that ductoscopic cytology is not sufficiently sensitive for the diagnosis of malignancy and therefore we have developed an intraductal biopsy device that obtains microbiopsies under direct visualisation [19]. The limitations of cytology can be also overcome by the development of novel technologies such as image analysis (IA), molecular diagnostics and real-time optical biopsy [22,23]. The scoring system for endoscopic appearances suggested earlier in this study may help the dicision-making process in clinical practice and aid communication between clinicians. This proposed system requires future validation in large studies correlating the macroscopic appearances of lesions with their final histological diagnosis. It would aid, and not substitute, histological examination, especially in some cases where histology is not definitive.

Our study shows that MD is technically feasible in most patients and has a potential in the early detection of breast cancer. Furthermore, MD can complement other investigations in diagnosing DCIS. In one patient in this study, PND was associated with DCIS that was not detected by mammography, ultrasound, discharge cytology, MRI or galactography. The role of MD in guiding breast-conserving surgery (BCS) in some cases of DCIS has sparked some controversy in the recent years. Dooley WC [24] used routine intra-operative MD during lumpectomy in 201 patients. Of the 201 patients (16 with atypical ductal hyperplasia, 52 with ductal carcinoma-in-situ, and 133 with stage 1 or 2 breast cancers), 150 (74.6%) could be successfully dilated and scoped. MD reduced the incidence of positive margins from 23.5% to only 5.0%. The author concluded that routine operative MD reduced the need for re-excision surgery and found substantially more cancerous and precancerous disease than anticipated by routine preoperative mammography and ultrasound. Conversely, Kim et al. [25] prospectively evaluated 30 patients undergoing therapeutic partial mastectomy for in-situ and invasive breast carcinoma. Although the number of patients in this trial was small, the authors concluded that MD could identify intraductal abnormalities during partial mastectomy in a significant number of patients. Nevertheless, many of these findings were either benign or within the standard field of resection, thus adding no benefit to the patient. Therefore, randomized controlled trials are needed to validate the hypothesis that MD can guide breast-conserving surgery (BCS) and reduce the need for re-excision procedures, especially in patients with DCIS.

The above discussion also raises important questions regarding the potential role of MD as a screening tool for breast cancer. To date there has been no studies examining the hypothesis that MD has a role in breast cancer screening. It is likely that MD will complement new evolving screening modalities such as ductal lavage cytology using microcatheters for screening and guiding risk-reducing strategies in high-risk population [2,5,7]. There is an apparent need for a large prospective clinical trial to evaluate the potential role of MD in the early detection of breast cancer.
